# *GmDAD1*, a Conserved *Defender Against Cell Death 1* (*DAD1*) From Soybean, Positively Regulates Plant Resistance Against *Phytophthora* Pathogens

**DOI:** 10.3389/fpls.2019.00107

**Published:** 2019-02-08

**Authors:** Qiang Yan, Jierui Si, Xiaoxia Cui, Hao Peng, Maofeng Jing, Xin Chen, Han Xing, Daolong Dou

**Affiliations:** ^1^Institute of Industrial Crops, Jiangsu Academy of Agricultural Sciences/Jiangsu Key Laboratory for Horticultural Crop Genetic Improvement, Nanjing, China; ^2^Department of Plant Pathology, Nanjing Agricultural University, Nanjing, China; ^3^Department of Crop and Soil Sciences, Washington State University, Pullman, WA, United States; ^4^National Center for Soybean Improvement, Nanjing Agricultural University, Nanjing, China

**Keywords:** *Glycine max*, *Phytophthora* resistant, *defender against apoptotic death 1* (*DAD1*), programmed cell death (PCD), ER stress

## Abstract

Initially identified as a mammalian apoptosis suppressor, defender against apoptotic death 1 (DAD1) protein has conserved plant orthologs acting as negative regulators of cell death. The potential roles and action mechanisms of plant DADs in resistance against *Phytophthora* pathogens are still unknown. Here, we cloned *GmDAD1* from soybean and performed functional dissection. *GmDAD1* expression can be induced by *Phytophthora sojae* infection in both compatible and incompatible soybean varieties. By manipulating *GmDAD1* expression in soybean hairy roots, we showed that *GmDAD1* transcript accumulations are positively correlated with plant resistance levels against *P. sojae*. Heterologous expression of *GmDAD1* in *Nicotiana benthamiana* enhanced its resistance to *Phytophthora parasitica*. *NbDAD1* from *N. benthamiana* was shown to have similar role in conferring *Phytophthora* resistance. As an endoplasmic reticulum (ER)-localized protein, GmDAD1 was demonstrated to be involved in ER stress signaling and to affect the expression of multiple defense-related genes. Taken together, our findings reveal that *GmDAD1* plays a critical role in defense against *Phytophthora* pathogens and might participate in the ER stress signaling pathway. The defense-associated characteristic of *GmDAD1* makes it a valuable working target for breeding *Phytophthora* resistant soybean varieties.

## Introduction

As sessile organisms, plants are continually exposed to various biotic and abiotic stresses. Therefore, complex stress perception, signal transduction and adaptation strategies have evolved in plants to cope with adverse environmental conditions. In particular, the programmed cell death (PCD) pathway has been demonstrated to play key roles in plant responses to both abiotic and biotic stresses (Dickman et al., 2001; Lam et al., 2001; Williams et al., 2010). In plant defense against pathogens, PCD restricts microbe growth and spreading in host tissue by eliminating excessive damaged cells (Kimchi, 2007).

Several PCD repressors have been identified in plants, including Bax inhibitor 1 (BI-1), B-cell lymphoma2 (Bcl-2)-associated athanogene (BAG), ER-luminal binding immunoglobulin protein (BiP), and defender against apoptotic death 1 (DAD1) (Gallois et al., 1997; Matsumura et al., 2003; Doukhanina et al., 2006; Williams et al., 2010; Jing et al., 2016; Li et al., 2016a,b). These repressors may increase or decrease plant resistance to different pathogens (Kawai-Yamada et al., 2004, 2009; Babaeizad et al., 2009; Watanabe and Lam, 2009; Eichmann et al., 2010; Ishikawa et al., 2011).

Among these PCD repressors, DAD1 is unique as it is conserved from yeast to mammals (Nakashima et al., 1993). Initially identified in a temperature-sensitive mutant hamster tsBN7 cell line, DAD1 is a subunit in the oligosaccharyltransferase (OST) complex, which is a core component for catalyzing *N*-glycosylation in ER (Yan et al., 2005; Peristera and Stephen, 2012). *N*-glycosylation is the attachment of oligosaccharides to certain asparagine residues of specific nascent proteins, which ensures their successful folding and export from ER. In *Drosophila melanogaster*, *DmDAD1* is essential for efficient *N*-glycosylation in developing tissues (Zhang et al., 2016). Disruption of *DmDAD1* increases accumulation of unfolded or misfolded proteins, which triggers stress signaling in ER and initiates PCD. In contrast, its overexpression stabilizes or increases *N*-glycosylation (Zhang et al., 2016).

Different hypotheses have been proposed for the roles of *DAD1* in maintaining cell viability. DAD1 may facilitate the targeting of OST complex to proteins directly responsible for cell viability. On the other hand, since DAD1 interacts with Mcl1, a Bcl2-family protein acting as an apoptosis inhibitor (Makishima et al., 2000), DAD1 may also affect cell viability in an OST-independent manner.

Plant *DAD1* orthologs from *Arabidopsis thaliana* and rice can rescue hamster tsBN7 cells from apoptosis (Gallois et al., 1997; Tanaka et al., 1997), which indicates they may also function as cell death repressors. Subsequent studies demonstrate that *AtDAD1* protects *Arabidopsis* protoplast cells against ultraviolet-C-induced PCD (Danon et al., 2004) and *DAD1* expression in *Gladiolus* decreases drastically during petal senescence (Yamada et al., 2004). Regarding the roles of *DAD1* proteins in plant defense, Wang X. J. et al. (2011) reported that *TaDAD2*-silenced wheat leaves have attenuated resistance to *Puccinia striiformis* with down-regulated expression of several defense-related genes. However, how this protein modulates plant-pathogen interactions has not been well characterized overall.

In this study, a *DAD1* orthologous gene was identified from soybean (*Glycine max*). Spatial and temporal expression of *GmDAD1* upon *P. sojae* infection, as well as its protein subcellular localization, were investigated. The function of *GmDAD1* in conferring *Phytophthora* resistance was dissected in soybean hairy roots with *GmDAD1* specifically silenced by RNAi, and *Nicotiana benthamiana* transgenic lines overexpressing *GmDAD1* or suppressing native *NbDAD1*. Our findings demonstrate that *GmDAD1* plays a critical role in *Phytophthora* resistance probably via regulating ER stress signaling.

## Materials and Methods

### Plant Materials and Growth Conditions

Two soybean varieties were used in this research: Williams 82 carrying the gene *Rps*1k, which confers resistance to *P. sojae* race 2 (Bernard and Cremeens, 1988) and Williams which does not carry any known *Rps* resistance gene (Bernard and Lindahl, 1972). Seeds of Williams 82 and Williams were sown in small plastic pots containing disinfected soil and maintained in greenhouse at 25°C and 16h:8h light/dark photoperiod. *N. benthamiana* plants were grown under identical conditions as described above.

### Culture of *Phytophthora* Pathogens

*Phytophthora sojae* isolates P6497 and P6497-RFP, which is a *P. sojae* strain constitutively expressing red fluorescence protein (RFP) (Xiong et al., 2014) were routinely cultured on 10% V8 juice agar plates at 25°C in the dark. *Phytophthora parasitica* was grown under the same conditions.

### *P. sojae* Inoculation and Soybean Samples Collection

Root, stem and leaf samples of the soybean varieties Williams 82 and Williams were collected at seedling and pod-filling stages. Hypocotyl inoculation of *P. sojae* was performed on Williams 82 and Williams plants as described previously (Sun et al., 2014). Agar disks containing hyphae were cut from fresh cultures and inoculated onto hypocotyl incision. After inoculation, the seedlings were placed in growth chamber to keep moisture. Inoculated stems were collected at 0, 6, 12, 24, and 48 h post inoculation (hpi). All samples were frozen immediately in liquid nitrogen and stored at -70°C. Three biological replicates were performed for each time point.

### DNA and RNA Extraction and RT-qPCR

Following supplier instructions, all DNA and RNA samples were extracted using the Hi-DNAsecure plant kit and the RNA simple Total RNA kit (Tiangen, China), respectively. For RNA samples, elimination of genomic DNA contamination and reverse transcription were performed using the HiScript II Q RT SuperMix reagent Kit (Vazyme, China).

qPCR reactions were performed on an ABI PRISM 7500 real-time PCR system (Applied Biosystems, United States) using the ChamQ^TM^ SYBR qPCR Master Mix reagent (Vazyme, China). Relative gene expression levels were calculated using the comparative 2^-ΔΔCT^ method (Livak and Schmittgen, 2001). Statistical analysis was conducted using the Student’s *t*-test with Excel 2010 software and the data were considered statistically significant for *P* < 0.05. qPCR primers for *GmDAD1* were designed from its conserved region. *PsTEF* (GenBank ID EU079791) was selected for determining *P. sojae* biomass (Yan et al., 2014). *GmCons4* (GenBank ID BU578186.1) was selected as endogenous reference in soybean (Libault et al., 2008). *NbEF1a* (GenBank ID AY206004) was used as *N. benthamiana* reference in the VIGS (virus-induced gene silencing) assay.

Defense-related genes analyzed in this research include five pathogenesis-related (PR) genes: *PR1a*, *PR2*, *PR3*, *PR4* and *PR5* (Bertini et al., 2003; Chen et al., 2007; Mazarei et al., 2007; Maldonado et al., 2014); the JA-regulated defense gene *plant defensin 1.2* (*PDF1.2*) (Lorenzo and Solano, 2005); the ethylene (ET) signaling marker gene *ethylene response factor 1* (*ERF1*) (Lorenzo et al., 2003); the reactive oxygen species (ROS) biosynthetic gene *NADP oxidase* (*NADPHOX*) and two ROS scavenging genes: *catalase* (*CAT*) and *ascorbate peroxidase* (*APX*) (Perez and Brown, 2014). We employed the sequences of *G. max* if the genes have been reported already, or obtained them by searching in the soybean EST and genome databases^1^ using orthologous sequences from *A. thaliana* as queries. All primers were designed using the Primer Premier 5 software. Primer specificity was evaluated by sequence similarity comparison and melting curve results of RT-qPCR. The primers of ER related genes were designed used the same strategy. The analyzed ER-stress related genes were the *binding immunoglobulin protein* (*Bip*), the *protein disulphide isomerase* (*PDI*), the *calnexin1* (*CNX1*), the *ER lumen-localized Dnaj protein3a* (*ERdj3A*), the *luminal binding domain/glucose-regulated protein 94* (*GRP94*), the *basic region/leucine zipper motif 17* (*bZIP17*) and the downstream gene *vacuolar processing enzyme* (*VPE*) (Rojo et al., 2004; Cai et al., 2014; Tiziana and Roberto, 2014). All primers used in this study and detailed information were listed in Supplementary Table S1.

### Subcellular Localization of the GmDAD1 Protein

For subcellular localization, the full-length coding sequence (CDS) of *GmDAD1* was amplified from cDNAs of the Williams variety using primer pair pBIN-G-DAD-F/R (Supplementary Table S1). The 351-bp *GmDAD1* CDS was then translationally fused with GFP after cloning into pBIN-GFP (Zhang et al., 2014) using *Kpn*I and *Xba*I sites. After sequencing validation, *GmDAD1-GFP* and *mCherry-HDEL* constructs were introduced into *Agrobacterium* tumefaciens stain GV3101. The two *Agrobacterium* liquid cultures were mixed and co-infiltrated into *N. benthamiana* leaves using a blunt syringe. After maintained for 48 h in greenhouse, agroinfiltrated leaves were detached and visualized with a laser scanning confocal microscope (Zeiss, GERMANY) at 488 and 591 nm for GFP and mCherry detection, respectively.

### Plasmid Construction for Soybean Cotyledon Transformation

The pBIN-GFP-*GmDAD1* construct which was used to determine GmDAD1 subcellular localization was also used to overexpress *GmDAD1* in soybean hairy roots, and the pBIN-GFP empty vector was used as control which allows expression of the *GFP* only. To make the *GmDAD1*-RNAi construct, partial *GmDAD1* gene was amplified (using primers p12-DAD-F and p12-DAD-R) and cloned into pDONR221 (Invitrogen, United States) and then entered in pHellsGate12:GFP via Gateway LR reaction. Modified from pHellsGate12 (Wesley et al., 2001), pHellsGate12:GFP harbors a 35S:*GFP*:nos expression cassette (Yan et al., 2014). After sequence validation, the pBIN-GFP-*GmDAD1*, *GmDAD1*-RNAi, the empty pBIN-GFP and pHellsGate12:GFP vectors were introduced into *Agrobacterium rhizogenes* strain K599 by electroporation.

### Plasmid Construction for *N. benthamiana* Transformation

To overexpress *GmDAD1* in *N. benthamiana*, the full length of *GmDAD1* CDS was obtained from cDNAs of the Williams variety using primer pair pDONR-DAD-F/R (Supplementary Table S1) and then cloned into the entry vector pDONR221 via Gateway BP reaction. After sequencing validation, the fragment was then entered in pEarlyGate202 via LR recombination reaction between the entry clone and the destination vector (Invitrogen, United States) (Earley et al., 2006). To make Tobacco Rattle Virus (TRV)-based VIGS construct targeting *NbDAD1*, partial fragment of *NbDAD1* was amplified using primer pair TRV:NbDAD-F/R and cloned into pTRV2 (Liu et al., 2002) using *Kpn*I and *EcoR*I sites. All constructs were validated by sequencing and transformed into *A. tumefaciens* strain EHA105 for *N. benthamiana* transformation and GV3101 for VIGS experiment.

### Soybean Cotyledon Transformation

Surface-sterilized soybean seeds were soaked in sterilized water overnight and then germinated on medium containing 0.5% sucrose and 1.2% agar in growth chamber with 16h:8h light/dark photoperiod. About 5 days after germination, unblemished cotyledons were harvested for *A. rhizogenes*-mediated transformation. Transformation was performed as described previously (Yan et al., 2014). After about 3 weeks of cultivation, transformed hairy roots became abundant at inoculated cotyledons. Positive transformants were selected by detecting GFP signal under fluorescence microscopy, cut off from cotyledons, and cultivated on White medium (Supplementary Table S2) for further verification and resistance level test.

### *N. benthamiana* Transformation and Virus-Induced Gene Silencing (VIGS)

*Nicotiana benthamiana* plants overexpressing *GmDAD1* were generated via *A. tumefaciens* mediated leaf disk transformation (Horsch et al., 1985). The T1 seeds harvested from self-pollinated T0 plants were surface-sterilized with 70% ethanol for 30 s, and 10% sodium hypochlorite solution for 5 min, then washed by sterilized water for five times. The sterilized seeds were germinated on MS medium with 100 mg/L glufosinate ammonium (Sigma, United States). T2 seeds were collected and sown in small plastic pots. After 2 weeks, the seedlings were sprayed with 100 mg/L glufosinate ammonium solution. Resistant were transplanted to new pots and confirmed by both genomic DNA and cDNA PCR using gene-specific primers (DAD-Test-F/R). The T2 plants were used for functional characterization.

For TRV-VIGS assay, *Agrobacterium* cultures harboring pTRV1 and pTRV2-VIGS (TRV2-NbDAD1, TRV2 empty vector or TRV2-NbPDS used as positive control of silencing) were mixed and infiltrated into *N. benthamiana* leaves using a blunt syringe (Fu et al., 2002). Inoculated plants were maintained at 20°C in greenhouse for effective virus infection and spread.

### Resistance Assay of *N. benthamiana* Against *Phytophthora parasitica*

Leaves from 5 to 6-week-old *N. benthamiana* plants were detached and inoculated with 20 μl *P. parasitica* zoospores (10^4^ ml^-1^) per leaf. Inoculated leaves were then kept in a moist chamber and lesion diameters were measured at 36 and 60 hpi. Representative infected leaves were photographed at 60 hpi under a UV lamp and then stained with trypan blue to visualize the infected area. The experiment was repeated three times with similar results and at least 20 leaves were inoculated for each biological replicate. Two weeks after infiltration, leaves from TRV and NbDAD1-VIGS plants were inoculated with *P. parasitica* using the same strategy. Lesion diameters were measured at 36 and 48 hpi due to the semi-dwarf phenotype of NbDAD1-VIGS plants. At least 10 lesions per construct were measured with three biological repeats. Student’s *t*-test was used to analyze the significance of differences. Difference were considered as significant when *P* < 0.05.

### Root Infection and Observation

After verification by detection of GFP fluorescence and qPCR, transgenic hairy roots of similar length (approximately 3 cm) were excised and dipped in the zoospore suspension (10^4^ zoospores per ml) of *P. sojae* race P6497-RFR for 5 min as described previously (Xiong et al., 2014). Inoculated roots were placed in Petri dishes containing 0.6% agar in the dark at room temperature. At 12, 24, and 36 hpi, the infection progression was monitored under an OLYMPUS MVX10 (OLYMPUS, Japan) fluorescence microscope via RFP fluorescence detection at 535 nm. The *P. sojae*-specific gene *PsTEF* was used for qPCR quantification of the relative biomass of *P. sojae.* For each sample, about 10 infected hairy roots were collected and pooled for DNA/RNA extraction which helps to reduce bias and increase statistical accuracy (Graham, 1991; Subramanian et al., 2005; Graham et al., 2007).

### Western Blotting Assay

About 10 transgenic roots with GFP fluorescence were collected and ground in liquid nitrogen. Total proteins were extracted with the extraction buffer (50 mM Tris-HCl, pH 7.5, 5 mM EDTA, 2 mM DTT, 1% triton, 2% polyvinylpolypyrrolidone and Roche complete protein inhibitor tablets). The samples were boiled for 10 min in 6× sodium dodecyl sulfate (SDS) loading buffer. SDS-PAGE and immunoblotting were performed in a mini-gel apparatus and submarine gel transfer systems (Bio-Rad, United States), respectively. Proteins were then transferred onto polyvinylidene fluoride (PVDF) membranes and then membranes were blocked with 5% non-fat dry milk in 0.01 M PBST for 1 h and then incubated with anti-GFP (1:1,000) (Sigma, United States) for 2 h at room temperature. After washing by TBST three times, the membrane was incubated with IRDye^®^800CW Goat anti-rabbit IgG (LI-COR, United States) secondary antibody at room temperature for 1 h. Protein bands were detecting using the Odyssey^®^ CLx quantitative fluorescence imaging system (LI-COR, United States).

### Sequence Analysis and Alignment

The conserved and transmembrane domains of GmDAD1 were analyzed with InterProScan and TMPRED respectively (Hofmann and Stoel, 1993; Jones et al., 2014). Multiple sequence alignment was performed using the BioEdit software (Hall, 1999).

## Results

### ER-Located GmDAD1 Shares Conserved Regions With Other Plant DAD1 Orthologs

**FIGURE 1:**
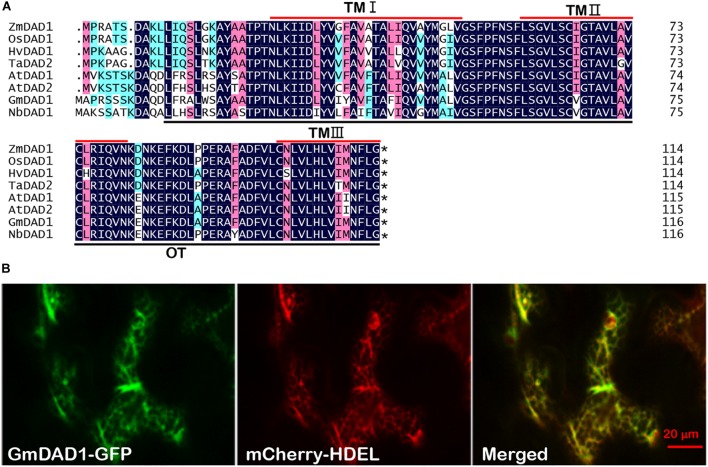
Molecular characterization and subcellular localization of GmDAD1 protein. **(A)** Sequence alignment of GmDAD1 and other defender against cell death (DAD) proteins. The darkblue (100%), pink (75%), and cyan (50%) boxes represent levels of amino acid identity or similarity. TM, transmembrane domain; OT, oligosaccharyltransferase domain. At, *Arabidopsis thaliana*; Hv, *Hordeum vulgare*; Zm, *Zea mays*; Os, *Oryza sativa*; Ta, *Triticum aestivum*. The asterisk indicates the stop codon. **(B)** Subcellular localization of GmDAD1 was performed via transient expression system in *Nicotiana benthamiana*. Green and red fluorescence represent the signal of GFP fusion protein and ER marker mCherry-HDEL, respectively. The reticulate fluorescence pattern of GmDAD1-GFP and its co-localization with mCherry-HDEL indicate accumulation in the ER.

*GmDAD1* (Gma.7542.2.S1_at) was identified from an Affymetrix Genechip microarray data analysis on soybean and *P. sojae* interaction (Zhou et al., 2009). *GmDAD1* was up-regulated in soybean varieties with different degrees of resistance to *P. sojae* (Zhou et al., 2009). Sequence analysis of *GmDAD1* (cloned from the Williams variety) revealed that its open reading frame (ORF) encodes a protein of 117 amino acid residues. GmDAD1 shares 91, 54, and 36% identities with DAD1 orthologs in *Arabidopsis thaliana*, *Homo sapiens*, and *Saccharomyces cerevisiae*, respectively. Similar to other plant DAD1 orthologs, GmDAD1 contains three transmembrane regions (residues 27–52, 61–81, and 95–115) and a subunit of OST (residues 13–116) (Figure 1A). To investigate the subcellular localization of GmDAD1, a *GmDAD1-GFP* fusion construct driven by the CaMV 35S promoter was expressed in *N. benthamiana* leaves. GmDAD1-GFP co-localized in the cytoplasm with mCherry-HDEL, an endoplasmic reticulum (ER) marker, demonstrating the ER localization of GmDAD1 (Figure 1B).

### *GmDAD1* Expression Is Induced Upon *P. sojae* Infection

**FIGURE 2:**
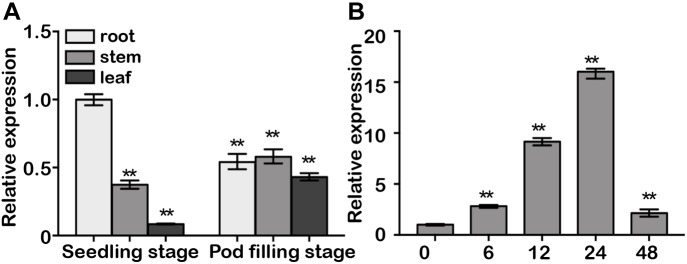
Gene expression analysis of *GmDAD1*. **(A)** *GmDAD1* mRNA levels in various tissues of soybean cultivar Williams. Leaves, roots, and stems were harvested from plants at the seedling and pod filling stage. **(B)** Expression profiles of *GmDAD1* in Williams (compatible interaction) at 0, 6, 12, 24, 48 h post inoculation (hpi) with *P. sojae* (P6497). The relative expression level was normalized to soybean *GmCons4* (GenBank: BU578186.1). Means and standard deviations were calculated from three independent biological replicates. Data were analyzed by using Student’s *t*-tests (^∗∗^*P* < 0.01).

*GmDAD1* transcript can be detected ubiquitously in roots, stems and leaves during plant development in cv Williams, with root being the organ exhibiting highest expression (Figure 2A). Interestingly, leaves showed much higher *GmDAD1* transcript accumulation at pod filling stage than seedling stage (Figure 2A). Similar *GmDAD1* expression pattern was detected in Williams 82 variety in the seedling stage (Supplementary Figure S1). On the contrary, the expression of *GmDAD1* is higher in roots at the pod filling stage in Williams 82 than in Williams.

After inoculation with P6497, a *P. sojae* isolate of race 2, the compatible variety Williams showed elevated *GmDAD1* expression which peaked at 24 hpi and subsequently decreased (Figure 2B). In the incompatible variety Williams 82, *GmDAD1* was also significantly induced by *P. sojae* infection at 24 hpi (Supplementary Figure S1).

### *GmDAD1* Enhances Resistance to *P. sojae* in Soybean Hairy Roots

**FIGURE 3:**
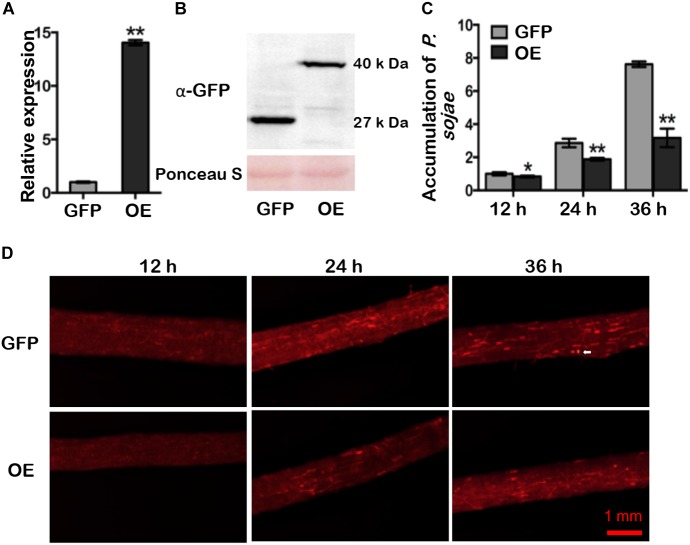
*GmDAD1* overexpression enhances resistance to *P. sojae* in soybean hairy roots. **(A)** Expression of *GmDAD1* in the *GmDAD1-GFP* overexpressing (OE) and control (*GFP*) hairy roots without *P. sojae* infection. Samples derived from different pooled root materials. Control has been transformed with the empty vector which allows expression of the GFP only. **(B)** Western blotting of proteins from hairy roots expressing GFP (control) and GmDAD1 fused with GFP tag. **(C)** Relative biomass of *P. sojae* determined by qPCR in inoculated OE and GFP hairy roots at 12, 24, and 36 hpi. Values represent the means of three replicates and 10 hairy roots were used for each biological replicate. Data were analyzed by using Student’s *t*-tests (^∗^*P* < 0.05, ^∗∗^*P* < 0.01 compared with the control). **(D)** Microscopic analysis of *P. sojae* colonization in infected soybean hairy roots. The OE and control GFP hairy roots were inoculated with zoospore suspension (10^4^ zoospore/ml) of the *P. sojae* P6497-RFP. Photos were taken at 12, 24, 36 hpi. The white arrow indicates a germinating oospore.

RT-qPCR analysis of ten mixed hairy roots displaying GFP fluorescence indicated that expression of *GmDAD1* in *GmDAD1-GFP* overexpression (OE) plants was nearly 14-fold higher that in the control (GFP) (Figure 3A). Western blotting also showed the accumulation of the GmDAD1-GFP fusion protein (Figure 3B). When OE and GFP hairy roots were inoculated with *P. sojae* P6497-RFP (Xiong et al., 2014), the biomass of *P. sojae* was significantly and consistently less in OE hairy roots than in GFP samples at 12, 24, and 36 hpi (Figure 3C). In the GFP control, the invasion hyphae emerged at 12 hpi, rapidly extended at 24 hpi, and almost filled the entire tissue at 36 hpi (Figure 3D). In contrast, hyphal growth was limited and the invasion hyphae were much sparser in *GmDAD1-GFP* overexpression roots (Figure 3D), which is consistent with the lower accumulation of *P. sojae* biomass (Figure 3C).

### Silencing of *GmDAD1* Reduces Resistance to *P. sojae* in Soybean Hairy Roots

**FIGURE 4:**
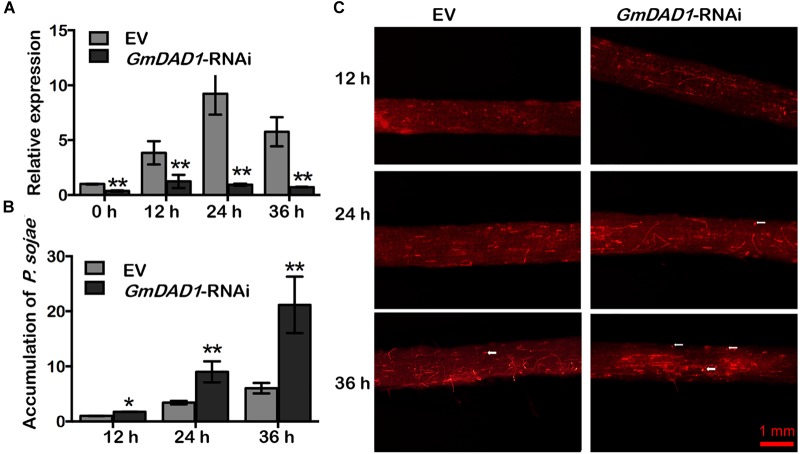
Silencing of *GmDAD1* reduces resistance to *P. sojae* in soybean hairy roots. **(A)** Relative expression of *GmDAD1* was determined by RT-qPCR in inoculated hairy roots in which *GmDAD1* was silenced via RNAi (*GmDAD1*-RNAi) or empty vector (EV) at 0, 12, 24, and 36 hpi. **(B)** Relative biomass of *P. sojae* was determined in inoculated hairy roots *GmDAD1*-RNAi or EV at 12, 24, and 36 hpi. Values represent the means of three replicates ± SD. Data were analyzed by using Student’s *t*-tests (^∗^*P* < 0.05, ^∗∗^*P* < 0.01 compared with the control). **(C)** Microscopic analysis of *P. sojae* colonization in soybean hairy roots. The control EV and *GmDAD1*-RNAi hairy roots were inoculated with zoospore suspension (10^4^ zoospore/ml) of the *P. sojae* P6497-RFP. Photos were taken at 12, 24, 36 hpi. The white arrows indicate germinating oospores.

RNAi-directed silencing of *GmDAD1* in soybean hairy roots (Figure 4A) was performed as described previously (Yan et al., 2014). Both *GmDAD1*-RNAi (RNAi) and EV control (EV) roots were inoculated with *P. sojae* P6497-RFP. Compared with control, *GmDAD1*-RNAi roots showed gradually increased *P. sojae* biomass accumulation over time (Figure 4B). Furthermore, a greater hyphal growth and higher oospore germination can be observed in *GmDAD1*-RNAi roots (Figure 4C). Our results indicated that *GmDAD1* is important for soybean resistance against *P. sojae*.

### *GmDAD1* Affects the Expression of Multiple Defense-Related Genes

**FIGURE 5:**
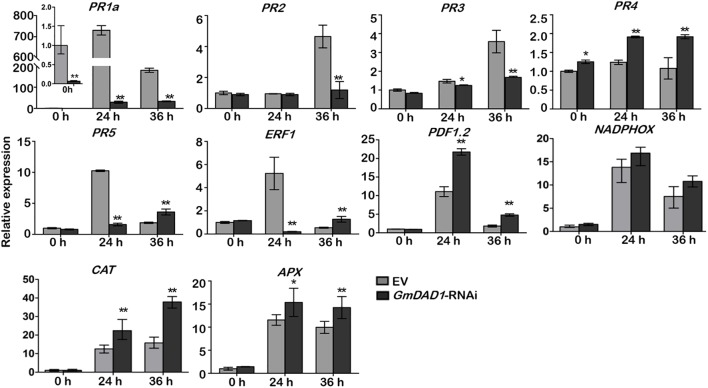
*GmDAD1* affects the expression of multiple defense-related genes. RT-qPCR analysis of the expression patterns of defense-related genes in the EV and *GmDAD1*-RNAi transgenic hairy roots after inoculation with *P. sojae*. Values represent the means of three replicates ± SD. Data were analyzed by using Student’s *t*-tests (^∗^*P* < 0.05, ^∗∗^*P* < 0.01 compared with the control).

To further determine whether the expression of defense-related genes was affected by *GmDAD1* silencing, we assessed the expression of several genes in hairy roots inoculated with *P. sojae*, including the marker genes of SA, and JA/ET signaling pathways, ROS generation and scavenging. The expression of *PR1a*, *PR2*, *PR3*, *PR5* and *ERF1* were decreased in *GmDAD1*-RNAi roots after *P. sojae* inoculation. It is to note that the expression of *PR1a* was also dramatically suppressed without inoculation (Figure 5). In contrast, the expression of *PDF1.2*, *PR4*, and two ROS scavenging genes, *CAT* and *APX*, were induced in the *GmDAD1* silencing roots infected with *P. sojae* (Figure 5). No significant change of *NADPHOX* expression was observed when *GmDAD1* was silenced (Figure 5).

### *GmDAD1* Is Involved in *P. sojae*-Activated ER Stress Signaling

**FIGURE 6:**
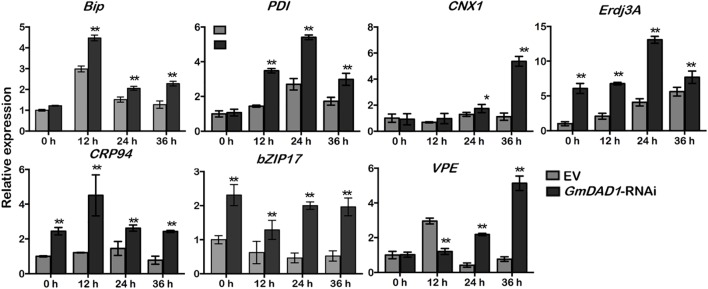
*GmDAD1* is involved in *P. sojae*-activated ER stress signaling. Expression patterns of ER stress-related genes in the EV and *GmDAD1*-RNAi transgenic hairy roots after inoculation with *P. sojae* at 0, 12, 24, 36 hpi. Values represent the means of three replicates ± SD. Data were analyzed by using Student’s *t*-tests (^∗^*P* < 0.05, ^∗∗^*P* < 0.01 compared with the control).

Since DAD1 catalyzes the first step of protein N-linked glycosylation, disruption of *GmDAD1* is expected to trigger unfolded protein response (UPR), which facilitates proper protein folding in ER via inducing the expression of a series of relevant genes (Li et al., 2011). After *P. sojae* inoculation, the transcript accumulations of six UPR marker genes were examined in soybean hairy roots, including *Bip*, *PDI*, *CNX1*, *ERdj3A*, *GRP94*, and *bZIP17*. All these genes are induced at the onset of ER stress and mark the activation of adaptive UPR. Expression changes of *VPE* were also monitored since its protein product possesses caspase-1-like activity and acts downstream of UPR and is part of the ER-PCD pathway. Compared to EV control, *GmDAD1*-RNAi roots showed significantly higher transcript accumulations of all seven UPR/ER stress marker genes at both 24 and 36 hpi (Figure 6). *VPE* was upregulated at 12 hpi and its expression decreased at 24 and 36 hpi in EV hairy roots, On the contrary, different trend was observed in *GmDAD1* silencing hairy roots. The expression increased continuously through the selected time course, and was significantly higher at 24 and 36 hpi (Figure 6).

### *GmDAD1* Enhances Resistance to *P. parasitica* in *N. benthamiana*

**FIGURE 7:**
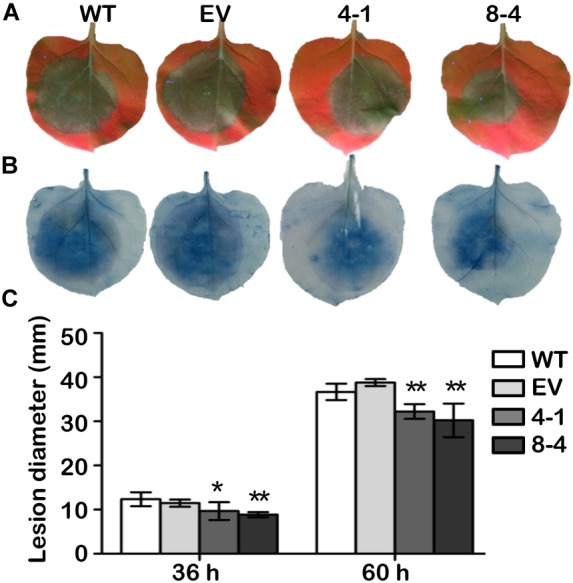
*GmDAD1* enhances resistance to *P. parasitica* in *N. benthamiana*. **(A)** Detached leaves from wild type (WT), empty vector control (EV) and *GmDAD1* overexpression plants (4-1 and 8-4) were inoculated with *P. parasitica* zoospores. Photographs were taken at 60 hpi under a UV lamp. **(B)** Trypan blue staining of the *P. parasitica* inoculated *N. benthamiana* leaves. **(C)** Lesion diameter of inoculated leaves measured at 36 and 60 hpi. The lesion size was calculated from 20 leaves ± SD with three biological repeats. Data were analyzed by using Student’s *t*-tests (^∗^*P* < 0.05, ^∗∗^*P* < 0.01 compared with the control).

To test whether *GmDAD1* confers resistance against other *Phytophthora* pathogens, transgenic *N. benthamiana* plants overexpressing *GmDAD1* were generated and verified (Supplementary Figure S2). Compared to wild-type (WT) and empty vector controls (EV) both *GmDAD1* overexpression lines tested (4-1 and 8-4) showed reduced disease symptoms (Figure 7A,B) and significantly smaller lesion diameters on leaves (Figure 7C) when infected with *P. parasitica* zoospores. The results suggest that *GmDAD1* overexpression enhances *N. benthamiana* resistance against *P. parasitica*.

### Silencing of *NbDAD1* in *N. benthamiana* Reduces Resistance to *P. parasitica*

**FIGURE 8:**
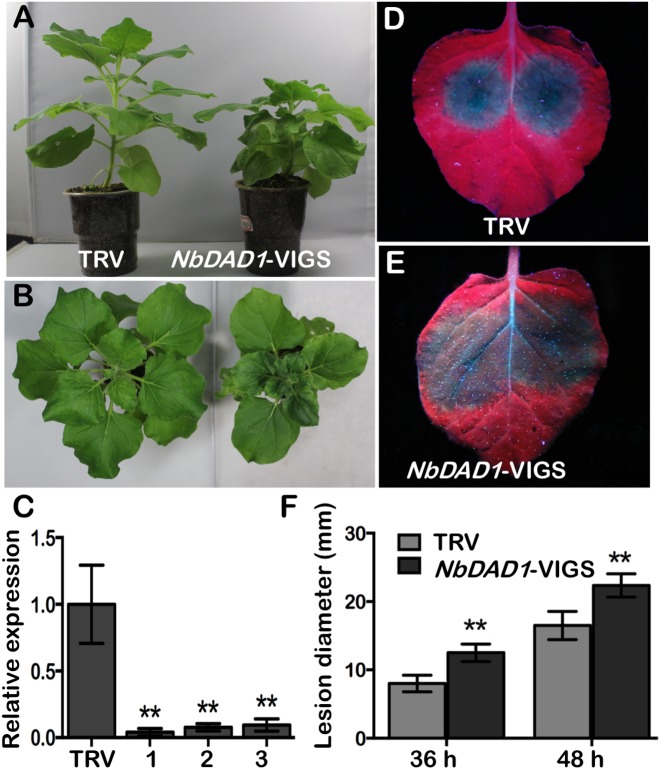
Silencing of *NbDAD1* in *N. benthamiana* reduces resistance to *P. parasitica*. **(A,B)** Side- and top- view of the TRV-infected control and *NbDAD1* silencing plants. **(C)** Relative expression of *NbDAD1* in *N. benthamiana* plants inoculated with TRV and three *NbDAD1* knock-down plants. The samples were collected 2 weeks after infection. *N. benthamiana NbEF1*α gene was used as reference for normalization. Data are the means ± SD calculated from three replicates. ^∗∗^*P* < 0.01. **(D,E)** Leaf phenotypes of TRV control and *NbDAD1*-VIGS after *P. parasitica* zoospores inoculation. Pictures were taken under UV illumination at 48 hpi. The experiments were repeated three times with similar results and representative images are shown. **(F)** Average lesion size of *NbDAD1* knock-down leaves and TRV control after *P. parasitica* zoospores inoculation. Averages were calculated from at least 10 lesions per construct. Data are the means ± SD. Data were analyzed by using Student’s *t*-tests (^∗∗^*P* < 0.01).

Since plant DADs are highly conserved, the native *NbDAD1* in *N. benthamiana* was silenced via TRV-based VIGS system for functional analysis. Compared to TRV-infected controls, plants infiltrated with *TRV-NbDAD1* displayed a semi-dwarf phenotype with increased branching (Figure 8A,B), which implies a possible role of *NbDAD1* in modulating growth and development. Three verified *NbDAD1* knock-down lines and TRV-infected controls were challenged with *P. parasitica* zoospores on detached leaves (Figure 8C). Silencing of *NbDAD1* led to significantly larger lesion diameters at both 36 and 48 hpi (Figure 8D–F), which indicates that *NbDAD1* is similar as *GmDAD1* in the function of conferring resistance against *P. parasitica*.

## Discussion

Being one of the most important crops worldwide, soybean can be infected by several major diseases, including the *Phytophthora* stem and root rot caused by *P. sojae* (Tyler, 2007). Continual efforts have been made to characterize novel defense genes against *Phytophthora* pathogens (Sugimoto et al., 2012). Here we identified GmDAD1, an ER-membrane protein from soybean, and dissected its function in plant–*Phytophthora* interactions.

Being evolutionary conserved across plant and animal species, DAD1 is a subunit of the OST complex, which catalyzes the first step of protein N-linked glycosylation in ER (Kelleher and Gilmore, 1997; Sanjay et al., 1998). In both animals and plants, the expression of *DAD1* orthologs responds to a wide range of adverse environmental stimuli, including injury (Zhu et al., 2008), temperature (Lee et al., 2003), and pathogen infection (Wang X. J. et al., 2011). *DAD1* inhibits undesired cell death triggered by host defense.

*N*-glycosylation has been reported to play a critical role in plant–pathogen interactions. For example, site-mutation on the *N*-glycosylation motif of *A. thaliana* receptor kinase EFR bleaches its ligand binding and results in oxidative burst elicitation capacity resulting in higher susceptibility of the plant to bacterial pathogens (Haweker et al., 2010). Several reports on the role of DAD proteins in plant defense have been published so far. The Arabidopsis *dad1* mutant shows reduced secretion of PR proteins and resistance against pathogens (Wang et al., 2005). In wheat, knock-down of *TaDAD2* suppresses the expression of *PR1*, *PR2*, and *PR5* in response to the infection of *Puccinia striiformis* f. sp. *tritici* (Wang X. J. et al., 2011). We hence propose that *GmDAD1* may also play a role in soybean disease resistance.

In soybean, *GmDAD1* expression can be induced by *P. sojae* infection in both compatible and incompatible varieties, which indicates that *GmDAD1* serves as a non-specific defense gene to some extent. However, *GmDAD1* has consistently higher expression after *P. sojae* inoculation in the incompatible variety Williams 82, and its expression does not drop dramatically afterward at 48 hpi, as it happens in the compatible variety Williams. Therefore, *GmDAD1* may be subjected to distinct transcriptional regulations in *P. sojae* compatible and incompatible soybean varieties.

Since *GmDAD1* has highest transcript accumulation in roots, we adopted the soybean hairy root infection system for *P. sojae* resistance test. *GmDAD1* gain- and loss-of-function mutants exhibit opposite *P. sojae* resistance phenotypes, which indicates that *GmDAD1* contributes to the resistance of soybean against *P. sojae*. Similarly, knock-down of *NbDAD1*, the native *DAD1* ortholog in *N. benthamiana*, reduces plant resistance to another *Phytophthora* pathogen, *P. parasitica*. Heterologous expression of *GmDAD1* in *N. benthamiana* enhances resistance to *P. parasitica*. Our results reveal that *DAD1* is a potential valuable defense gene against *Phytophthora* pathogens and this disease resistance function is conserved across plant species.

Phytohormone signaling, which is mediated by SA during biotrophic and hemibiotrophic plant–pathogen interactions and JA and ET for necrotrophic plant pathogens, plays important roles in plant resistance (Glazebrook, 2005). Previously studies demonstrated that the resistance to *P. sojae* is mediated by the SA and ET signaling pathways (Moy et al., 2004; Sugano et al., 2014). Therefore, we assessed the expression of several key defense related genes by RT-qPCR. When *GmDAD1* silencing hairy roots were inoculated with *P. sojae*, the transcription of *PR1a*, *PR2*, *PR3*, *PR5*, and *ERF1* were significantly reduced. Since the PR genes are generally regarded as early markers of resistance response, the suppressed expression of these genes may be responsible for the compromised resistance at the begin of the infection process (from 0 to 24 hpi). Moreover, the two JA-dependent signal marker genes *PDF1.2* and *PR4* were up-regulated after *P. sojae* infection in the silenced hairy roots (later than 24 hpi). We inferred that this JA resistance signaling activation might be lately induced, and the up-regulation might be caused by the antagonistic effect of JA and SA pathways.

Reactive oxygen species are important messenger molecules in defense signal regulation. The expression of ROS-generating gene *NADPHOX* showed no difference between EV and *GmDAD1*-RNAi hairy roots, however, the ROS-scavenging genes *CAT* and *APX* were statistically significant up-regulated after *P. sojae* infection in the silencing roots, this means that the ROS signaling was not completely affected by *GmDAD1* silencing.

AS a core subunit of OST complex, DAD1 plays an important role in protein *N*-glycosylation (Peristera and Stephen, 2012), the defeat of protein *N*-glycosylation cause accumulation of misfolded proteins in ER and subsequently ER stress (Li et al., 2011; Cai et al., 2014). In soybean hairy roots infected by *P. sojae*, we found that *GmDAD1* acts as a repressor for multiple UPR marker genes. In detail, all tested genes become up-regulated at later stages of the infection when *GmDAD1* is silenced, indicating severe ER stress. We believe that this situation is caused by a less efficient or delayed defense signaling transduction. However, whether the suppression of defense-related genes was directly caused by the ER stress due to *GmDAD1* silencing need to be further investigated.

Under extreme condition such as pathogen infection, a prolonged ER stress is known to eventually activate the ER-PCD pathway. *Phytophthora* pathogens are hemibiotrophic. They initially establish a biotrophic relationship with their hosts, and switch to necrotrophic phase later than 15 hpi (Enkerli et al., 1997). In EV hairy roots, a sharp increase of *VPE*, a cystein proteinase mediating PCD via the maturation and activation of vacuolar proteins, was observed at 12 hpi most likely to limit and overcome the biotrophic phase of *P. sojae* infection. In *GmDAD1*-RNAi roots, *VPE* expression was relatively suppressed at the same infection stage, suggesting the failure of PCD induction. However, elevated expression of *VPE* was detected at 24 and 36 hpi indicating a later activation of ER-PCD pathway. This late apoptosis overlaps with the necrotrophic phase of *P. sojae*, which may be one of the reasons of the increased *P. sojae* accumulation in *GmDAD1* silencing hairy roots.

Disruption of *DAD1* causes growth defect or even embryonic lethality in animal systems (Brewster et al., 2000; Zhang et al., 2016). In this study, we have observed significantly reduced transformation rate when silencing *GmDAD1* in soybean hairy roots (Supplementary Figure S3). Moreover, knock-down of *NbDAD1* by VIGS caused a semi-dwarf phenotype in *N. benthamiana*. These results suggest that *DAD1* may play a similar role of regulating growth in plants most likely by acting on the *N*-glycosylation pathway of key proteins involved in plant development.

## Conclusion

We observed that GmDAD1, a conserved component of the OST complex, via participating in the ER-PCD and UPR pathways and affecting the expression of multiple defense-related genes, confers resistance to *Phytophthora* pathogens. Moreover, *GmDAD1* regulates plant growth and development likely by the effect on the *N*-glycosylation pathway. Taken together, *GmDAD1* can be considered as a promising target for the molecular breeding of *Phytophthora*-resistant soybean varieties.

## Author Contributions

DD and QY designed the project. QY, JS, and XaC performed the experiments and analyzed the data. XnC, HX, and DD guided the experimental work. DD, QY, HP, and MJ wrote the manuscript. All authors read and approved the final manuscript.

## Conflict of Interest Statement

The authors declare that the research was conducted in the absence of any commercial or financial relationships that could be construed as a potential conflict of interest.

## Footnotes

1 https://www.soybase.org/GlycineBlastPages/
