# Supplementary material for: GmDAD1, a Conserved Defender Against Cell Death 1 (DAD1) From Soybean, Positively Regulates Plant Resistance Against Phytophthora Pathogens
Source: Front Plant Sci. 2019 Feb 8;10:107. doi: 10.3389/fpls.2019.00107 (PMC6376896; doi:10.3389/fpls.2019.00107)
Supplement: Supplementary file 1 [file Table_1.docx]

**TABLE S1: Sequences of the gene-specific primer pairs used in the study**

| Primer name | Primer sequence | annealing temperature (^o^C) | target gene/ GenBank ID |  |
| --- | --- | --- | --- | --- |
| qNbDAD1-F | 5'-GGTAAACAAAGAAAACAAGGAA-3' | 47 | Nbv5.1tr6225149 | |
| qNbDAD1-R | 5'-CCAAATAGAGCACCAAAT-3' | 49 |  |  |
| qDAD1-F | 5'-CGCTTTCATCCAGGTTGTTT-3' | 50 | Glyma.14G201900 | |
| qDAD1-R | 5'-AGAACAAAATCCGCAAAAGC-3' | 48 |  |  |
| TRV:NbDAD-F | 5'-GGGGTACCGAAATCTTCAGCGACCAA-3' | 61 |  | |
| TRV:NbDAD-R | 5'-CGGAATTCGATTCGGAGACAAACAGC-3' | 60 |  | |
| pBIN-G-DAD-F | 5'-CGGGGTACCATGGCTCCTCGGTCTAGC-3' | 67 |  | |
| pBIN-G-DAD-R | 5'-GCTCTAGATTAACCAAGGAAGTTCATGATCAC-3' | 61 |  | |
| p12-DAD-F | 5'-GGGGACAAGTTTGTACAAAAAAGCAGGCTGCTCCTCGGTCTAGCAGC-3' | 72 |  | |
| p12-DAD-R | 5'-GGGGACCACTTTGTACAAGAAAGCTGGGTACCAAGGAAGTTCATGATCAC-3' | 71 |  | |
| pDONR-DAD-F | 5'-GGGGACAAGTTTGTACAAAAAAGCAGGCTTCACCATGGCTCCTCGGTCTAGCAGCAA-3' | 74 |  | |
| pDONR-DAD-R | 5'-GGGGACCACTTTGTACAAGAAAGCTGGGTTTTAACCAAGGAAGTTCATGATCAC-3' | 71 |  | |
| DAD-Test-F | 5'-ATGGCTCCTCGGTCTAGCAGC-3' | 58 |  | |
| DAD-Test-R | 5'-TTAACCAAGGAAGTTCATGATC-3' | 49 |  | |
| Cons4-F | 5'-GATCAGCAATTATGCACAACG-3' | 50 | BU578186.1 | |
| Cons4-R | 5'-CCGCCACCATTCAGATTATGT-3' | 52 |  |  |
| PsTEF1-F | 5'-TGATCGTGCTGAACCACCC-3' | 53 | EU079791 | |
| PsTEF1-R | 5'-CGAGCGACGGTCCATCTT-3' | 53 |  |  |
| NbEF1α-F | 5'-AGAGGCCCTCAGACAAAC-3' | 50 | AY206004 | |
| NbEF1α-R | 5'-TAGGTCCAAAGGTCACAA-3' | 46 |  |  |
| GmPR1a-F | 5'-TGATACGGTTGCTGCTTA-3' | 46 | Glyma.13G252400 | |
| GmPR1a-R | 5'-CATCTGTGCCACTTAGTTC-3' | 49 |  |  |
| GmAPX1-F | 5'-TTCGGAACCATCAAGCAC-3' | 48 | Glyma.U021900 | |
| GmAPX1-R | 5'-GGTAGAAATCGGCGTAGC-3' | 50 |  |  |
| GmCAT1-F | 5'-CAACCCCAAGTCCCACAT-3' | 50 | Glyma.17G261700 | |
| GmCAT1-R | 5'-ACACCGAAGCCATCCATA-3' | 48 |  |  |
| GmPR5-F | 5'-CGAATCGTGGAACATCAG-3' | 48 | Glyma.11G025600 | |
| GmPR5-R | 5'-AAGAACACCGTCGCAGTC-3' | 50 |  |  |
| NADPHOX-F | 5'-GACCTTAGTGTTGGGAGT-3' | 48 | Glyma.08G018900 | |
| NADPHOX-R | 5'-AAATAAGCGTTGGTAGTCT-3' | 45 |  |  |
| GmERF1-F | 5'-ATTTCATCACCGTCGTCC-3' | 48 | Glyma.18G252300 | |
| GmERF1-R | 5'-TAGCCACTTCAACATTCCAC-3' | 50 |  |  |
| GmPDF1.2-F | 5'-GCAAACTGAGGCAAAGAC-3' | 48 | Glyma.13G278000 | |
| GmPDF1.2-R | 5'-GCACCAACAGCGAAAATC-3' | 48 |  |  |
| qGmPDI-F | 5'-TTGGTTGAAGGCGTACAAGGATGG-3' | 57 | Glyma.04G247900 | |
| qGmPDI-R | 5'-ACTCCAGCAGAACATTCTTCCCAG-3' | 57 |  |  |
| qGmVPE-F | 5'-AACCCAAGGCCTGGAGTCAT-3' | 54 | Glyma.14G092800 | |
| qGmVPE-R | 5'-TCGCCGGTGTAATCCTTTG-3' | 51 |  |  |
| qGmCNX1-F | 5'-AGCCAGATGACTGGGATGAG-3' | 54 | Glyma.04G202900 | |
| qGmCNX1-R | 5'-GCTTCCCATTCACCATCCTC-3' | 54 |  | |
| qGmERdj3A-F | 5'-GGATTTGGCTTGGATGA-3' | 45 | Glyma.18G204000 | |
| qGmERdj3A-R | 5'-TTGGCAGAACTCTTGGAC-3' | 48 |  | |
| qGmGRP94-F | 5'-AATCCAAAGGAGGTTAC-3' | 42 | Glyma.17G258700 | |
| qGmGRP94-R | 5'-TAGGTGGTACAAACAGG-3' | 45 |  | |
| qGmbzip17-F | 5'-GCACTCGCCCTCACCTGAT-3' | 55 | Glyma.03G123200 | |
| qGmbzip17-R | 5'-CTGTGCTTGGTAGCACTTCC-3' | 54 |  | |
| qGmBip-F | 5'-AGAAGGCTATGGAAGATGC-3' | 49 | Glyma.08G025700 | |
| qGmBip-R | 5'-GCCACATCCAGGAGAAGG-3' | 53 |  | |
| qGmIRE1a-F | 5'-GCCAACTCCATTACCTCTT-3' | 49 | Glyma.09G197000 | |
| qGmIRE1a-R | 5'-TCTTCCCTGATTTCCGTGT-3' | 49 |  | |
| qGmIRE1b-F | 5'-TAATCCTCCATGACGCTCAC-3' | 52 | Glyma.11G087200 | |
| qGmIRE1b-R | 5'-CCAGTTTTCGCATCCACTT-3' | 49 |  | |
| qGmPR2-F | 5'-TCCACTGCCATTGATACTG -3' | 49 | Glyma.03G132700 | |
| qGmPR2-R | 5'-ACTACCACCGAAGGAGACC -3′ | 53 |  | |
| qGmPR3-F | 5′-GCACTTGGTCTGGATTTG-3′ | 48 | Glyma.02G042500 | |
| qGmPR3-R | 5′- GGCTTGATGGCTTGTTTC -3′ | 48 |  | |
| qGmPR4-F | 5′- TGGAAACACCGAAGAATAC-3' | 47 | Glyma.19G245400 | |
| qGmPR4-R | 5′- TCCCAAGTTGAGCAATAAG-3' | 47 |  | |

**TABLE S2:** **Medium formulation of white medium.**

| Constituent | Concentration (mg/L) |
| --- | --- |
| KNO3 | 80.0 |
| Ca(NO3)2 | 287.0 |
| MgSO4 | 738.0 |
| Na2SO4 | 53.0 |
| KCl | 65.0 |
| NaH2PO4 | 19.1 |
| C6H8O7 | 2.0 |
| Thiamine | 0.1 |
| Pyroxidine | 0.1 |
| Nicotinic Acid | 0.5 |
| Glycine | 3.0 |
| MnSO4 | 6.6 |
| ZnSO4 | 2.7 |
| KI | 0.75 |
| H3BO3 | 1.5 |
| Sucrose | 20000 |
| Gelrite | 3800 |
