# Supplementary figures and images for: GmDAD1, a Conserved Defender Against Cell Death 1 (DAD1) From Soybean, Positively Regulates Plant Resistance Against Phytophthora Pathogens
Source: Front Plant Sci. 2019 Feb 8;10:107. doi: 10.3389/fpls.2019.00107 (PMC6376896; doi:10.3389/fpls.2019.00107)

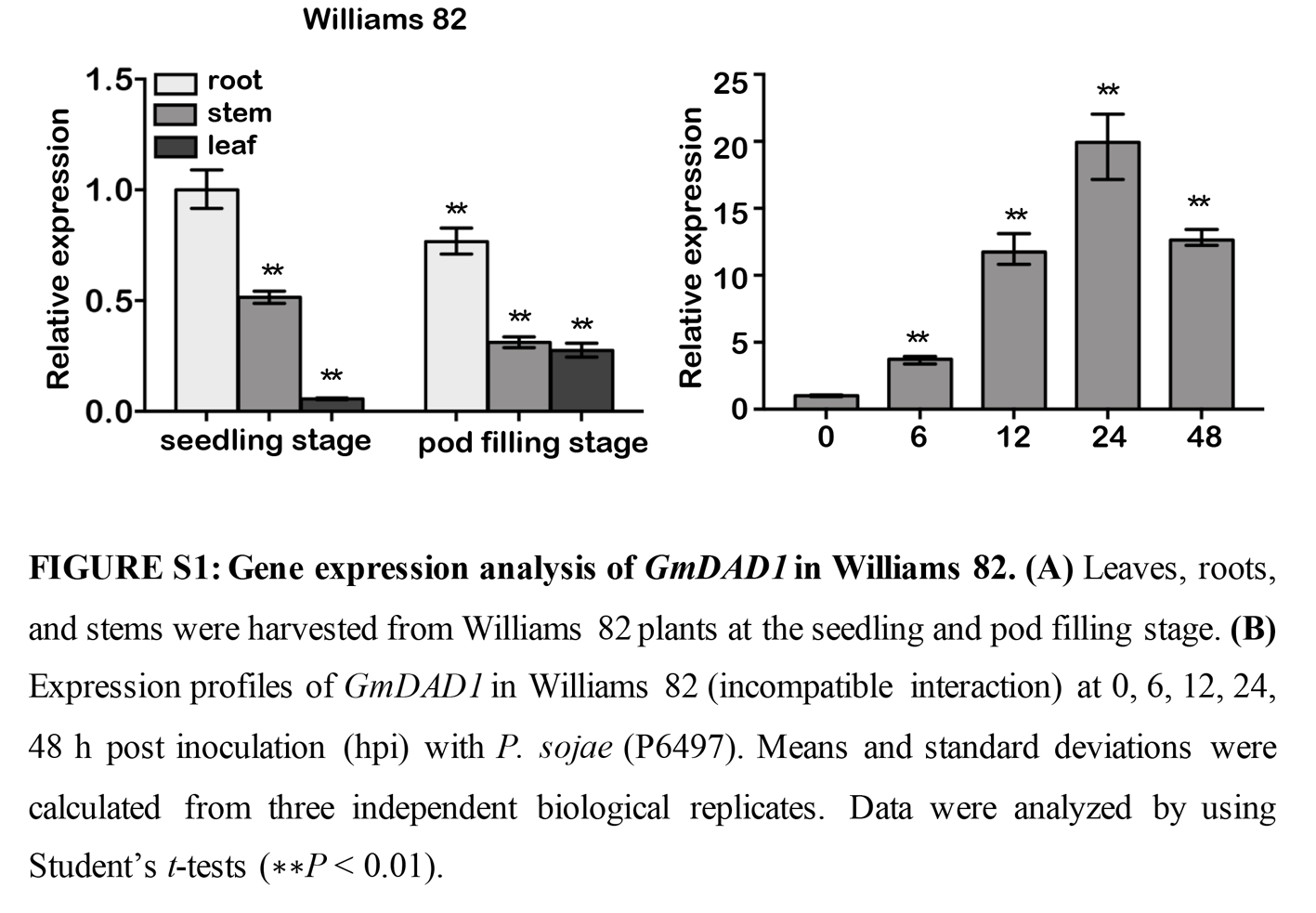

Supplement: Supplementary file 2 [file Image_1.tif]

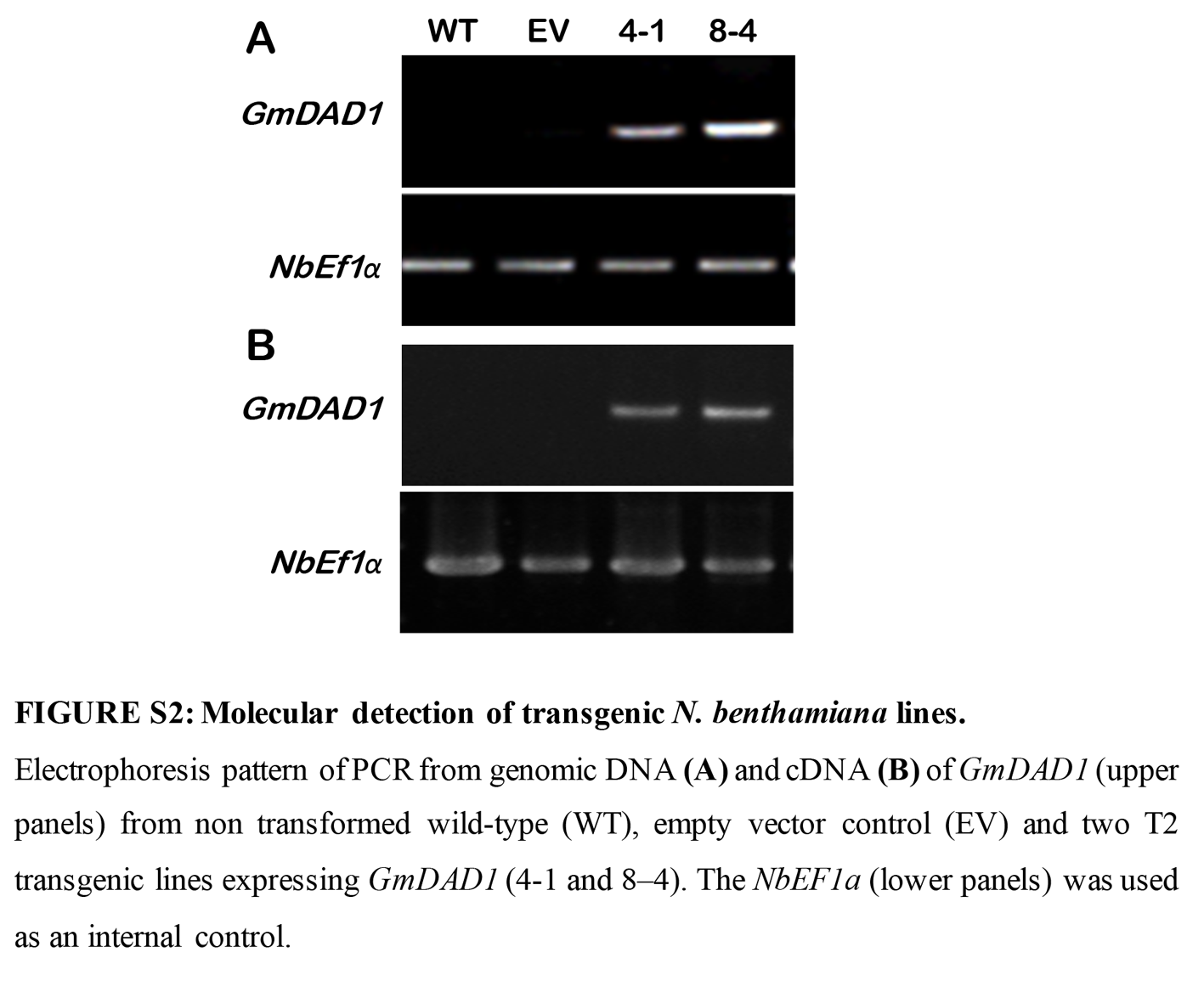

Supplement: Supplementary file 3 [file Image_2.tif]

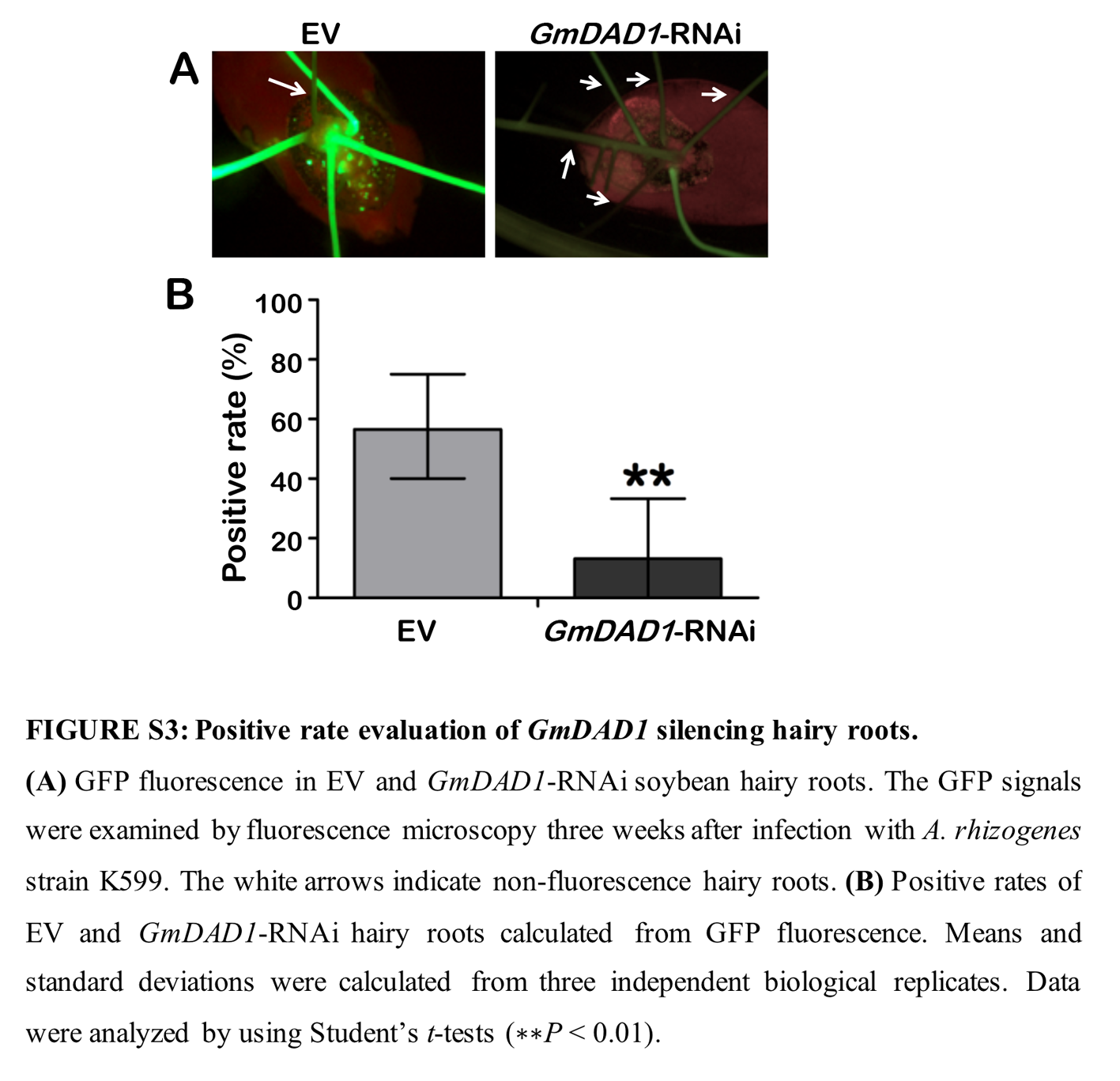

Supplement: Supplementary file 4 [file Image_3.tif]
